# Clinical characteristics and follow-up of benign convulsions with mild gastroenteritis among children

**DOI:** 10.1097/MD.0000000000014082

**Published:** 2019-01-11

**Authors:** Xiaohong Ma, Shaoyong Luan, Yiming Zhao, Xiumin Lv, Ruiyun Zhang

**Affiliations:** Department of Qingdao Municipal Hospital, Qingdao, Shandong, China.

**Keywords:** convulsion, gastroenteritis, norovirus, rotavirus

## Abstract

Benign convulsions with mild gastroenteritis have a high incidence in the North China, previous reports it has been caused by rotavirus infection, which is a non-febrile convulsion. In recent years, we observed that norovirus infection gradually increased all the years round and most of them are febrile convulsion.

Retrospective analysis was performed on 55 pediatric patients with benign convulsions with mild gastroenteritis (CWG) who were admitted between January 2010 and January 2015.

The median age of the norovirus infection group was greater than that of the rotavirus infection group. Norovirus infection has no obvious seasonality. The peak age of benign CWG onset was less than 3 years (74.5%). In 69.1% of all cases, the temperature was less than 38 °C. In 70.9% of cases, children had experienced more than 2 episodes. In 96.4% of all cases, convulsive episodes were shorter than 5 min. Convulsions mostly occurred within the first 2 days (78.2%) after gastroenteritis tract symptoms. Most episodes featured generalized tonic-clonic seizure (87.3%). Serum Na+ levels were lower while other biochemical test results were normal. Follow-up 2 years attack again (16.8%), intelligence quotient was not reduced.

CWG febrile or afebrile occurs most frequently among young children. In addition to the rotavirus, the norovirus might also cause this syndrome. The prognosis is favorable and long-term anti-seizure treatment is considered to be unnecessary.

## 1 Introduction

Convulsion with mild gastroenteritis (CWG) was first reported in 1982 by the Japanese researcher Morooka.^[1]^ In 1995, more detailed diagnostic criteria were proposed by Komorie,^[2]^ as follows:

(1) the child was previously healthy;
(2) afebrile convulsions were accompanied by mild gastroenteritis, possible mild dehydration, absence of apparent acid intoxication, and electrolyte imbalance;
(3) convulsions mainly occurred during the winter season and the gastroenteritis might last for 1 to 5 days;
(4) convulsions might manifest as single or multiple episodes of generalized tonic-clonic seizure (GTCS);
(5) normal interictal electroencephalogram (EEG);
(6) normal serum electrolytes, serum glucose, and cerebrospinal fluid (CSF) with stool antigen test positive for rotavirus; and
(7) favorable prognosis with rare relapse and unimpeded development.

However, very few research was focused on the Chinese population, and there is a lack of data. Particularly in community hospitals, such repeated afebrile or low-fever convulsions are frequently misdiagnosed as complex febrile convulsion, viral encephalitis, toxic encephalopathy, or epilepsy, which can impede clinical treatment and lead to overtreatment. Additionally, some children with CWG do not meet the diagnosis criteria above. In the present study, we summarize and discuss cases of CWG over the past 5 years in our hospital.

## 2 Subjects and methods

### 2.1 Research subjects

We selected an observation group of 55 patients who were admitted to our hospital between January 2010 and January 2015 with CWG. The diagnosis of benign convulsions with mild gastroenteritis was made when a patient met both of the following criteria:

(1) seizures associated with gastroenteritis without clinical signs of dehydration or electrolyte derangement;
(2) a body temperature less than 38.0 °C before and after the seizures.

Among all of the cases, including 32 cases of male and 23 cases of female, age ranging from 4 months to 8 years old, 21 were positive for the stool rotavirus antibody, 7 were positive for the stool norovirus antibody, and 3 were positive for both antibodies. The same period 50 cases with mild gastrointestinal without convulsions formed the control group. In the members of the control group, 18 were positive for the stool rotavirus antibody, 8 were positive for the stool norovirus antibody, and 4 were positive for both antibodies. The members of the control group included 28 males and 22 females and aged between 5 months and 6 years old. Pure positive rotavirus was observed in 39 cases with the median age of 9 months. Pure norovirus positive was observed in 15 cases with the median age of 17 months, which is more than that of the rotavirus group. Different from rotavirus caused CWG that occurred only in fall and winter, the norovirus caused CWG can occur all year round. We examined the family history and disease history of the research subjects. In order to assess potential convulsion causes, we conducted routine stool tests, culture assays, and examinations including measurements of serum electrolytes and serum glucose, assessments of liver/kidney function, EEGs, and brain CT or MRI. After the parents signing the informed consent, we examined the CSF obtained via lumbar puncture. Relapse status and mental development were assessed by following up interviews conducted during clinic visits and telephone conversations with the parents or caregivers (once every 6 months). Wechsler Intelligence tests were conducted after 2 years at the onset.

1.3 Antigen tests for stool rotavirus and norovirus were performed using the colloidal gold method and immunochromatography, respectively. SPSS17.0 was used for data analysis.

## 3 Results

### 3.1 Patient age at onset of convulsions

The onset ages of convulsions were as follows: 41 cases were under 3 years old, 10 were 3 to 6 years old, and 4 were older than 6 years. The cases under 3 years old accounted for 74.5% of the total cases.

### 3.2 Clinical manifestations

The clinical manifestations of gastroenteritis tract symptoms were as follows: 38 cases had fever with body temperature under 38°C (69.1%), 45 experienced vomiting, and 48 experienced diarrhea. Among these patients, 4 had a family history of febrile convulsions and 1 had a family history of afebrile convulsions. None had a family history of epilepsy.

### 3.3 Gastroenteritis tract symptoms and convulsions

The intervals between gastroenteritis tract symptoms and convulsions were as follows: 26 cases had convulsions on day 1 of gastroenteritis tract symptoms, 17 on day 2, 6 on day 3, 3 on day 4, 1 on day 5, and 2 cases had conclusions before the gastroenteritis tract symptoms appeared. Overall, 78.2% of the cases had convulsions that occurred during the first 2 days.

### 3.4 Frequency of convulsions

That seizures occur in clusters ranging from 1 to 4 episodes with a 24-h period. The frequency of the convulsions was as follows: 16 cases had 1 episode of convulsions, 29 had 2 episodes, 8 had 3 episodes, and 2 had 4 episodes. In total, 70.9% of cases had more than 2 episodes.

### 3.5 Manifestations of convulsions

The manifestations of GTCS were 48 cases (87.3%); 5 cases experienced unusual staring behavior, and 2 cases experienced absence seizures.

### 3.6 Duration of convulsions

The convulsion durations were as follows: less than 1 min in 35 cases; 1 to 5 min in 18 cases; 6 to 10 min in 2 cases. Cases with convulsive durations of less than 5 min accounted for 96.4%.

### 3.7 Lab test results

The lab test results for the patients were as follows: all patients had normal stool results and negative stool culture, normal CSF (virus isolation and antibody tests were not performed owing to lab hardware limitations), normal brain CT and MRI results. Eighteen cases had abnormal EEG, and among these, 8 displayed diffuse or local slow waves, 6 had cerebral wave dysrhythmia or asymmetry, and 4 had θ or δ waves that did not match the norm for their age group.

### 3.8 Blood biochemistry comparisons

Blood biochemistry comparisons between the observation group and the control group are as follows: the observation group had lower sodium levels, and there were no other differences between the 2 groups. These data are shown in Table 1.

**Table 1:**
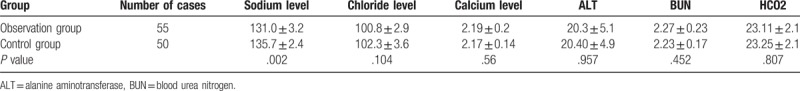
Biochemical test comparison between the 2 groups.

### 3.9 Treatment and follow-up

Those in the convulsion group received i.m. or i.v. injections of diazepam after the first occurrence and diazepam plus phenobarbital after the second occurrence. The dosages were as follows: diazepam 0.1 to 0.2 mg/kg to a maximum of 10 mg, and phenobarbital 5 to 8 mg/kg. Both groups received identical supportive treatment. In total, 70.9% of the cases had 2 convulsive episodes. Two cases had more than 3 episodes, and despite receiving i.v. Valproate, they relapsed. In follow-up 6 months to 2 years later, 33 person-times, again suffered from convulsion after Gastroenteritis tract symptoms in total of 197 person-times (16.8%). Wechsler preschool and Primary Scale of intelligence (<6 years); Wechsler Intelligence Scale for Children (>6 years). These 2 tests were conducted after 2 years at the onset, intelligence quotient was not associated with reduced compared with their peers.

## 4 Discussion

Gastroenteritis commonly occurs in children aged 6 to 24 months in Northern China during the cold season. This common illness results from the rotavirus infection. Accumulating knowledge and improved laboratory technology has indicated that Gastroenteritis tract infections with norovirus, sapovirus, adenovirus, and coxsakie virus are not rare.^[3,4]^ Areas in which children have been vaccinated for the rotavirus have a significantly increased frequency of norovirus infection. CWG is primarily caused by the virus infections listed above. Some reports^[5,6]^ have shown that 2% to 3% of rotavirus infections are accompanied with benign convulsions. The disease peaks in frequency among children aged 1 to 2 years old.^[7]^ Boys and girls have similar occurrence rates. Although some reports have indicated that girls have a higher occurrence rate than boys with a ratio of 1:1.5 to 1.8, our data showed that boys had a higher occurrence rate than girls, possibly owing to the higher hospitalization rate among boys in Northern China.

Convulsions associated with CWG are mainly manifested as GTCSs. Partial convulsions and limited convulsions have been shown to account for 13% to 65%^[2,5,8]^ of total convulsion occurrences. Episode frequency is generally between 1 and 8 times and individuals with more than 2 episodes account for 57% to 88% of total cases.^[5,8,9]^ The episode duration is usually between 30 sec and 5 min, although episodes lasting more than 30 min have also been reported.^[10]^ Convulsions often occur between day 1 and day 6 after the onset of Gastroenteritis tract symptoms. Occasionally, convulsions can occur before gastroenteritis tract symptoms.

The mechanisms of this condition are still unclear. As CWG only affects children, it was previously hypothesized to be related to the immature developing nervous system and the resultant susceptibility in young children, similar to febrile convulsions. Among cases of CWG, the most commonly detected virus is the rotavirus. The rotavirus can directly invade the central nervous system, causing cerebropathy, encephalitis, or convulsions.^[11]^ Indeed,^[12,13]^ children with rotavirus gastroenteritis have been found to have detectable rotavirus antibodies and RNA. Further research^[14]^ has confirmed that children with intestinal rotavirus infections accompanied by convulsions have detectable levels of rotavirus RNA. Additionally,^[15]^ dendrites in the central nervous systems of animals infected with the rotavirus have been confirmed to have detectable levels of NSP4. NSP4 is a glycosylated protein specific to the rotavirus that can induce the release of reserved intracellular calcium. This leads to a simultaneous influx of extracellular calcium, destroying the calcium homeostasis. Increased calcium causes the secretion of chloride ions. NSP4 can specifically destroy cellular junctions, thus causing membrane instability, resulting in diarrhea. The rotavirus replicates and induces NSP4 production in the central nervous system, thus causing nervous toxicity and neurotransmitter disequilibrium. Indeed, the rotavirus infection may activate a range of immune factors, therefore causing convulsions. Intestinal viruses invade peripheral blood and brain cells and activate the immune system. Such immune processes have immunoprotective anti-infective effects and help to maintain the physiological balance. However, super-reactivities resulting from immune disorders may also have harmful effects on the body, for instance, aggravating brain damage. Kwashima et al^[16]^ found that children with rotavirus gastroenteritis accompanied with convulsions had significantly elevated NO levels in the CSF, and that this elevation was much higher than that resulting from purulent meningitis, encephalitis, or febrile convulsions. Additionally, Zhang et al^[17]^ found that children with mild gastroenteritis accompanied with benign convulsions had significantly elevated IL-β levels in the serum and CSF. Levels of IL-β level are correlated with the convulsion magnitude, convulsion duration, and extent of brain damage.

Zifan et al^[18]^ tested serum sodium levels among children with CWG and found that some of them had hyponatremia, which would affect their convulsion duration. Motoyama et al^[19]^ also found that children with mild rotavirus gastroenteritis accompanied with convulsions had significantly low sodium and chloride levels, indicating that sodium ion channels are implicated in CWG. However, Kang et al^[5]^ showed that even though levels of serum sodium were decreased among children with convulsions, they were not significantly different from those observed in a non-convulsion group. Other researchers^[20]^ found that corrected serum calcium levels were significantly decreased, consistent with the pathological changes resulting from the rotavirus infection. In the present study, we showed that the observation group had significantly lower sodium levels when compared with the control group; however other ion levels were not different.

Our long-term observation indicated that children with CWG do not require anti-seizure treatment. CWG has a short duration, with most episodes ending within 24 hours; it therefore only requires acute phase treatment. In the present study, we used diazepam and phenobarbital to treat patients with more than 2 convulsive episodes. Those patients accounted for 70.9% of the total study subjects. Sodium valproate was used to treat 2 patients; however, the follow-up assessments indicated that these treatments were ineffective. Although individuals with CWG might experience several episodes of convulsions, the prognosis is favorable and mental development appears to continue in a normal way. Some researchers^[21]^ believe that benzodiazepine is ineffective; and propose that carbamazepine, a sodium ion channel, be used as an effective alternative, with a treatment duration of less than 1 week.

In summary, CWG is a common condition involving low febrile or afebrile convulsions in pediatric patients. In addition to the rotavirus the norovirus might also cause this syndrome. The age of children with norovirus infection is higher than that of rotavirus. CWG tends to occur among younger children, although it is also present among older children. Convulsive episodes mainly manifest as GTCSs, and occasionally, limited and partial seizures. Convulsions mostly occur between day 2 and day 3 after the onset of gastroenteritis tract symptoms, but can also occur before gastroenteritis tract symptoms. Episodes are generally short, but relapse is frequent. Although the disease mechanisms are unclear, the prognosis is favorable and long-term anti-seizure treatment is considered to be unnecessary.

## Author contributions

**Conceptualization:** Xiaohong Ma.

**Data curation:** Yiming Zhao.

**Formal analysis:** Xiumin Lv.

**Investigation:** Xiumin Lv, Ruiyun Zhang.

**Supervision:** Ruiyun Zhang.

**Writing – original draft:** Xiaohong Ma, Shaoyong Luan.

xiaohong ma orcid: 0000-0003-1271-3000.

## References

[1] Morooka K Convulsions and mild diarrhea. Shonika (Tokyo) 1982;23:131–7.

[2] Komori H Wada M Eto M Benign convulsions with mild gastroenteritis: a report of 10 recent cases detaliling clinical varieties. Brain Dev 1995;17:334–7. 8579220 10.1016/0387-7604(95)00074-l

[3] Kawano G Oshige K Syutou S Benign infantile convulsions associated with mild gastroenteritis: a retrospective study of 39 cases including virological tsts and efficacy of anticonvulsants. Brain Dev 2007;29:617–22. 17544607 10.1016/j.braindev.2007.03.012

[4] Chen SY Tsai CN Lai MV Norovirus infection as a cause of diarrhea-associated benign infantile seizures. Clin Infect Dis 2009;48:849–55. 19239351 10.1086/597256

[5] Kang B Kim DH Hong YJ Comparison between febrile and afebrile seizures associated with mild rotavirus gastroenteritis. Seizure 2013;22:560–4. 23642407 10.1016/j.seizure.2013.04.007

[6] Lloyd MB Lloyd JC Gestel PH Rotavirus gastroenteritis and seizures in young children. Pediatr Neurol 2010;42:404–8. 20472191 10.1016/j.pediatrneurol.2010.03.002

[7] Verrotti A Nanni G Agostinelli S Benign convulsions associated with mild gastroenteritis: a multicenter clinical study. Epilepsy Res 2011;93:107–14. 21146369 10.1016/j.eplepsyres.2010.11.004

[8] Lee EH Chung S A comparative study of febrile and afebrile seizures associated with mild gastroenteritis. Brain Dev 2013;35:636–40. 23111347 10.1016/j.braindev.2012.09.014

[9] Dura-Trave T Yoldi-Petri ME Gallinas-Victoriano F Infantile convulsions with mild gastroenteritis: a retrospective study of 25 patients. Eur J Neurol 2011;18:273–8. 20618844 10.1111/j.1468-1331.2010.03120.x

[10] Goldwater PN Rowland K Thesinger M Rotavirus encephalopathy: pathogenesis reviewed. J Paediatr Child Health 2001;37:206–9. 11328483 10.1046/j.1440-1754.2001.00596.x

[11] Fischer TK Ashley D Kerin T Rotavirus antigenemia in patients with acute gastroenteritis. J Infect Dis 2005;192:913–9. 16088842 10.1086/432549

[12] Sugats K Taniguchi K Yui A Analysis of rotavirus antigenemia and extraintestinal manifestations in children with rotavirus rotavirus gastroenteritis. Pediatrics 2008;122:392–7. 18676558 10.1542/peds.2007-2290

[13] Liu B Fujita Y Arakawa C Detection of rotavirus RNA and antigens in serum and cerebrospinal fluid samples from diarrheic children with seizures. Jpn J Infect Dis 2009;62:279–83. 19628905

[14] Tian P Ball JM Zeng CQ The rotavirus nonstructural glycoprotein NSP4 possesses membrane destabilization actibity. J Virol 1996;70:6973–81. 8794341 10.1128/jvi.70.10.6973-6981.1996 PMC190747

[15] Kawashima H Inage Y Ogihara M Serum and cerebrospinal fluid nitrite/nitrate levels in patients with rotavirus gastroenteritis induced convulsion. Life Sci 2004;74:1397–405. 14706570 10.1016/j.lfs.2003.08.014

[16] Zhang Y-D Gu F Xie H-Q The serum and cerebrospinal fluid interleukin 1 beta testing analysis among children in convulsions with mild gastroenteritis. Pediatr Emerg Med China 2014;8:522–3.

[17] Zifan E Alehan F Menascu S Clincal characterization of gastroenteritis-related seizure in children, impact of fever and serum sodlium levels. J Child Neurol 2011;26:1397–400. 21693651 10.1177/0883073811409222

[18] Motoyama M Ichiyama T Matsushige T Clinical characteristics of benign convulsions with rotavirus gastroenteritis. J Child NeurolV 24 2009;557–61. 10.1177/0883073808327829 19168832

[19] Yeom JS Kim Y-S Park JS Role of Ca^2+^ homeostasis disruption in rotavirus-associated seizures. J Child Neurol 2012;10:1–6. 10.1177/0883073812469052 23271755

[20] Ueda H Tajiri H Kimura S Clinical characteristics of seizures associated with viral gastroenteritis in children. Epilepsy Res 2015;109:146–54. 25524854 10.1016/j.eplepsyres.2014.10.021

[21] Capovilla G Verrotti A Infantile convulsions in association with mild gastroenteritis: an emerging clinical condition. Eur J Neurol 2011;18:203–4. 20629719 10.1111/j.1468-1331.2010.03140.x

